# Supplementary material for: Intracellular Aluminium in Inflammatory and Glial Cells in Cerebral Amyloid Angiopathy: A Case Report
Source: Int J Environ Res Public Health. 2019 Apr 24;16(8):1459. doi: 10.3390/ijerph16081459 (PMC6518255; doi:10.3390/ijerph16081459)
Supplement: Supplementary file 1 [file ijerph-16-01459-s001.pdf]

Supplementary Figure S1. Autofluorescence of the parietal lobe.

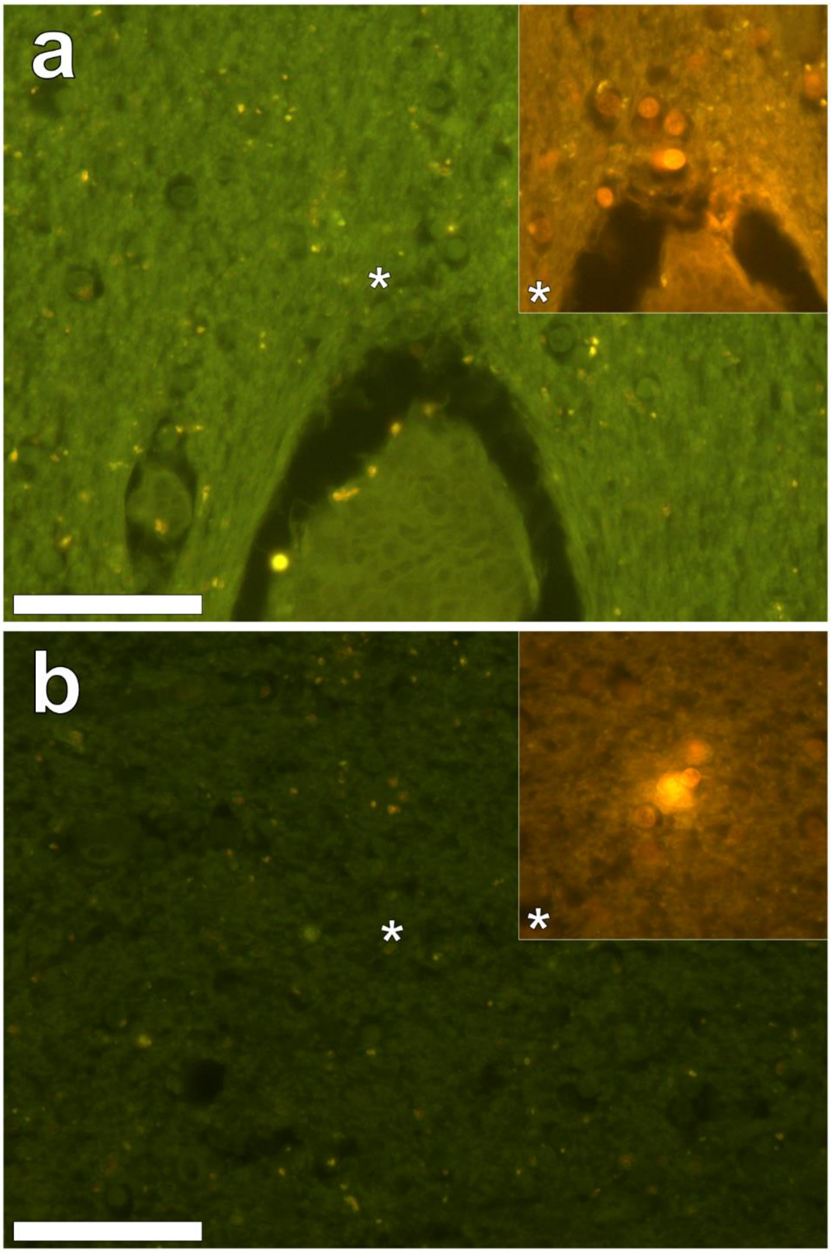

Supplementary Figure 1 Autofluorescence in white matter of the parietal lobe of a 59-year-old female donor with severe CAA, in the same region as lumogallion-reactive aluminium in an adjacent 5µm serial section. A green fluorescence emission was noted in the non-stained sections with lipofuscin evident as a yellow pigment. Inserts depict intracellular aluminium in glial-like cells surrounding vasculature **(a)** and in areas depicting cellular debris in lumogallion-stained serial sections (asterisks) **(b)**. Magnification X 400, scale bars: 50µm.

Supplementary Figure S2. Autofluorescence of the occipital lobe.

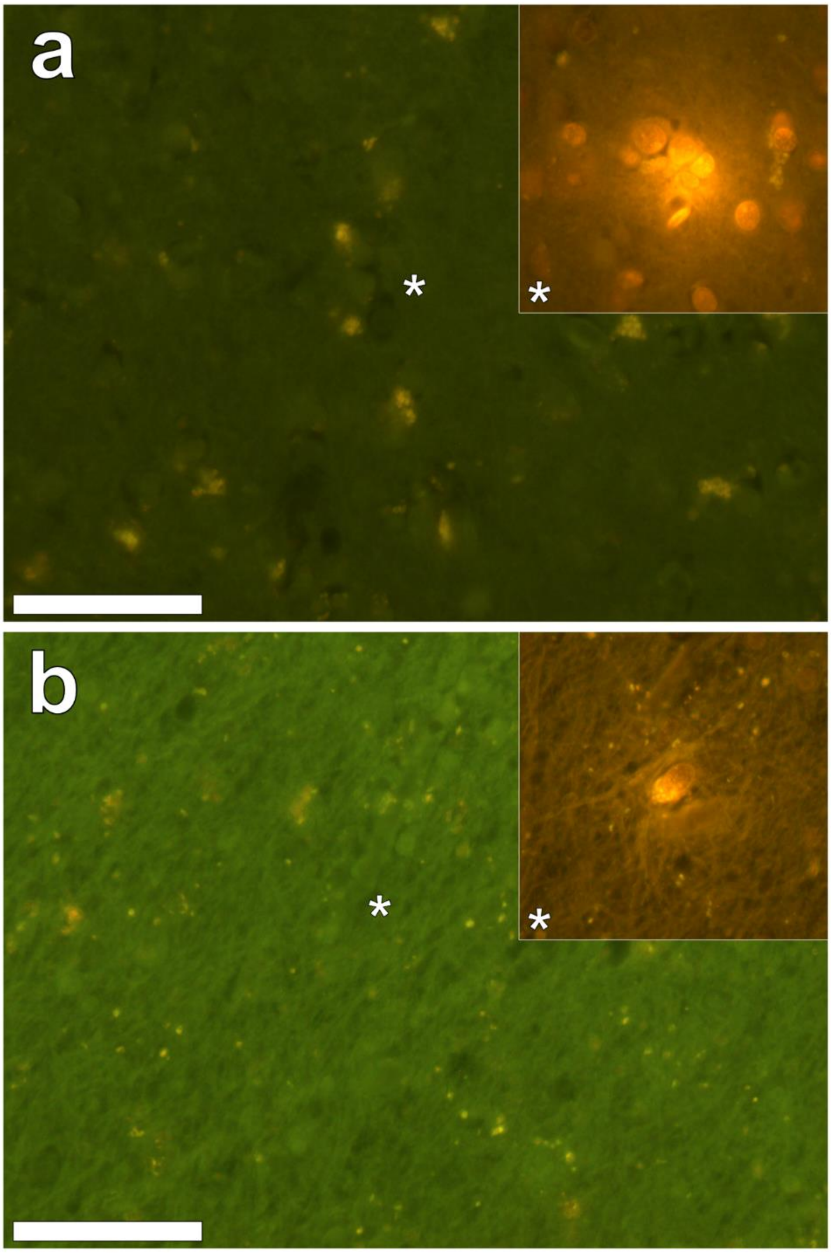

Supplementary Figure 2 Autofluorescence in grey and white matter of the occipital lobe of a 59-year-old female donor with severe CAA, in the same region as lumogallion-reactive aluminium in an adjacent 5μm serial section. A green fluorescence emission was noted in the non-stained sections with lipofuscin evident as a yellow pigment. Inserts depict intracellular aluminium in microglial-like cells surrounding astrocytes in grey matter **(a)** and in an astrocytic-like cell in white matter, in lumogallion-stained serial sections (asterisks) **(b)**. Magnification X 400, scale bars: 50μm.

Supplementary Figure S3. Autofluorescence of the temporal lobe.

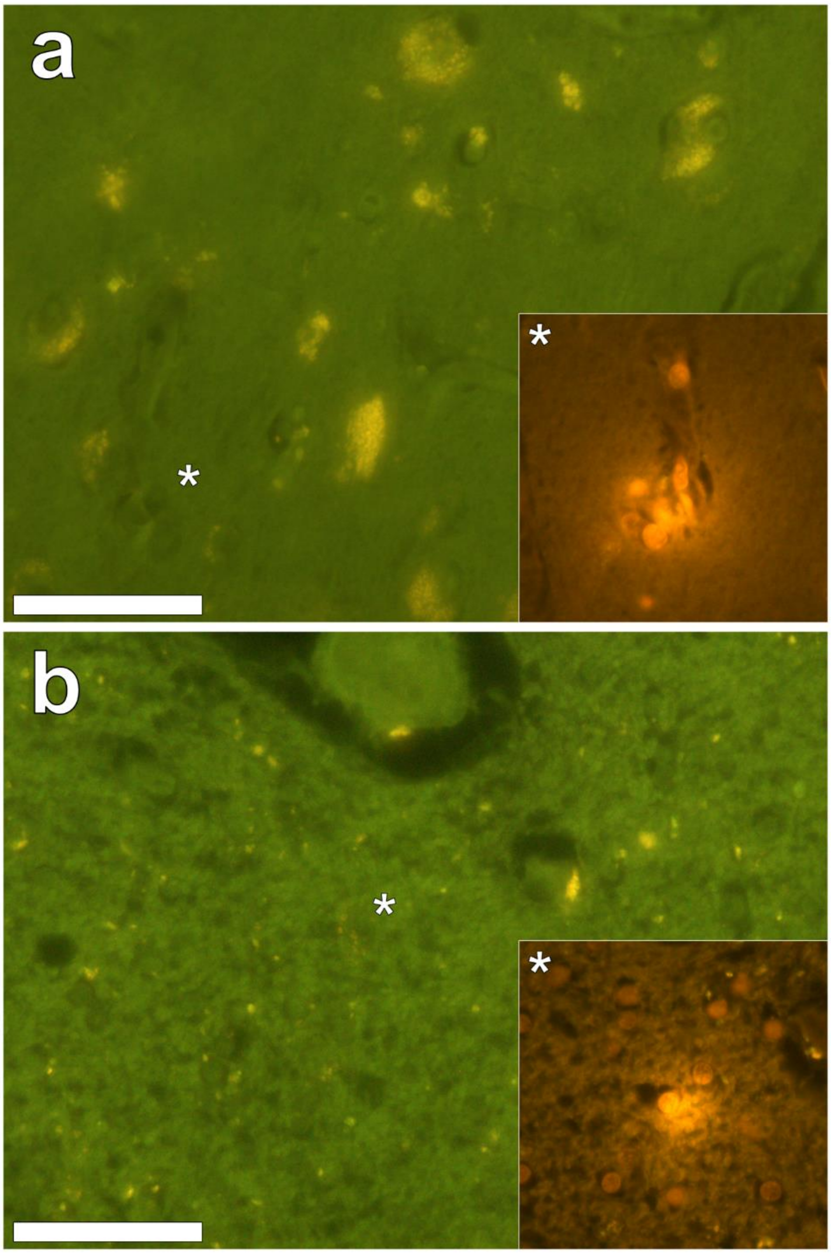

Supplementary Figure 3 Autofluorescence in grey and white matter regions of the temporal lobe of a 59-year-old female donor with severe CAA, in the same region as lumogallion-reactive aluminium in an adjacent 5µm serial section. A green fluorescence emission was noted in the non-stained sections with lipofuscin evident as a yellow pigment. Inserts depict intracellular aluminium in glial cells exhibiting astrocytic-like processes in grey **(a)** and white **(b)** matter regions in lumogallion-stained serial sections (asterisks). Magnification X 400, scale bars: 50µm.

Supplementary Figure S4. Autofluorescence of the hippocampus.

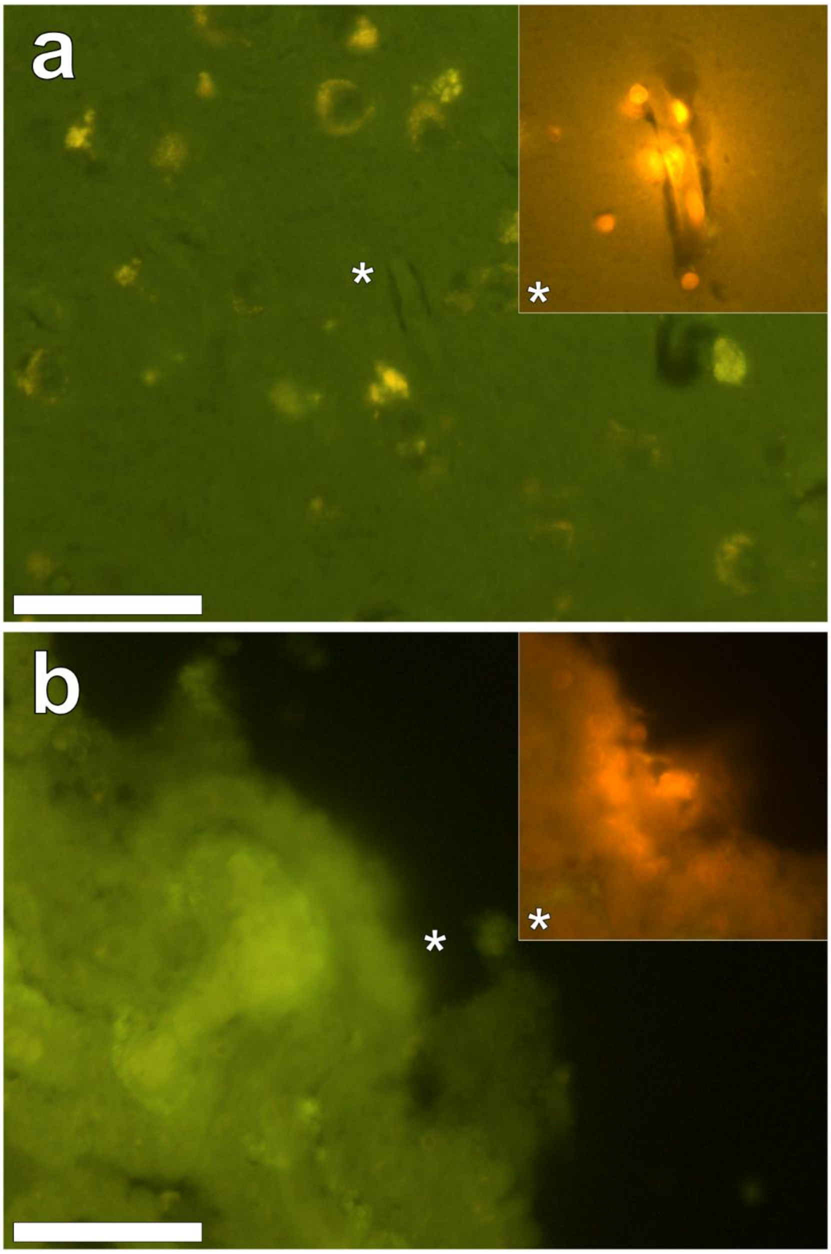

Supplementary Figure 4 Autofluorescence in the hippocampus of a 59-year-old female donor with severe CAA, in the same region as lumogallion-reactive aluminium in an adjacent 5µm serial section. A green fluorescence emission was noted in the non-stained sections with lipofuscin evident as a yellow pigment. Magnified inserts depict intracellular aluminium in inflammatory cells in the vessel wall **(a)** and within ependymal cells lining the choroid plexus **(b)** in lumogallion-stained serial sections (asterisks). Magnification X 400, scale bars: 50µm.
